# Conditional rotation of two strongly coupled semiconductor charge qubits

**DOI:** 10.1038/ncomms8681

**Published:** 2015-07-17

**Authors:** Hai-Ou Li, Gang Cao, Guo-Dong Yu, Ming Xiao, Guang-Can Guo, Hong-Wen Jiang, Guo-Ping Guo

**Affiliations:** 1 Key Laboratory of Quantum Information, CAS, University of Science and Technology of China, Hefei, Anhui 230026, China.; 2 Synergetic Innovation Center of Quantum Information and Quantum Physics, University of Science and Technology of China, Hefei, Anhui 230026, China.; 3 Department of Optics and Optical Engineering, University of Science and Technology of China, Hefei, Anhui 230026, China.; 4 Department of Physics and Astronomy, University of California, Los Angeles, California 90095, USA.

## Abstract

Universal multiple-qubit gates can be implemented by a set of universal single-qubit gates and any one kind of entangling two-qubit gate, such as a controlled-NOT gate. For semiconductor quantum dot qubits, two-qubit gate operations have so far only been demonstrated in individual electron spin-based quantum dot systems. Here we demonstrate the conditional rotation of two capacitively coupled charge qubits, each consisting of an electron confined in a GaAs/AlGaAs double quantum dot. Owing to the strong inter-qubit coupling strength, gate operations with a clock speed up to 6 GHz have been realized. A truth table measurement for controlled-NOT operation shows comparable fidelities to that of spin-based two-qubit gates, although phase coherence is not explicitly measured. Our results suggest that semiconductor charge qubits have a considerable potential for scalable quantum computing and may stimulate the use of long-range Coulomb interaction for coherent quantum control in other devices.

## Abstract (web summary)

Quantum-information processing requires gates that can operate on multiple qubits. Here, the authors demonstrate a controlled-NOT gate operation on two coupled charge qubits comprising electrons confined in semiconductor double quantum dots.

## Graphical abstract

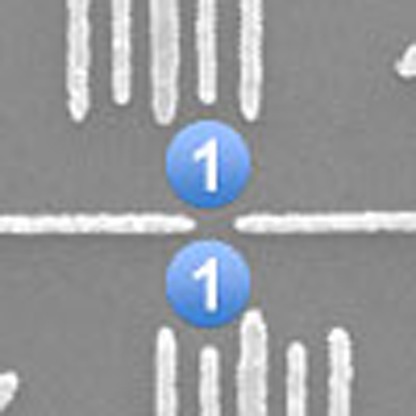

Semiconductor quantum dots (QDs), hailed for their potential scalability, are outstanding candidates for solid state-based quantum information processing^1^,^2^,^3^. Qubits, encoded by the charge occupancy of a single electron in a double QD, have attracted considerable attention^4^,^5^,^6^,^7^,^8^,^9^ for number of reasons. First, speed of gate operation is primarily determined by the inter-dot tunnelling rate, which can be made to be extremely fast. Second, initialization, manipulation and read-out are all intuitively simple in this all-electrical approach. Furthermore, the utilization of charge degree of freedom for quantum computation is compatible with today's mainstream information processing technology, and is suitable for scaling up to large-scale quantum circuits by taking advantage of the wealth of present semiconductor infrastructures.

One of the basic building blocks of universal quantum computation is a two-qubit gate. Previous research on two coupled semiconductor charge qubits have shown certain evidence of correlated oscillations^10^,^11^,^12^,^13^. However, the implementation of a two-qubit gate operation in QD charge qubits has not been demonstrated to date, largely owing to the technical challenges of achieving strong coupling between qubits and the ability to control gate pulses in the sub-nanosecond time scales.

In the following, we report the coherent manipulation of a capacitively coupled qubit pair. We achieve a strong electrostatic dipole coupling between two charge qubits. The large coupling energy enables us to completely and coherently turn on/off the Larmor oscillations of one qubit by pulse driving the charge on the other qubit. Based on this effect, we demonstrated a controlled-NOT (CNOT) operation, although phase coherence is not explicitly measured^14^,^15^,^16^,^17^,^18^,^19^. In addition, we combined this CNOT operation and universal single-qubit gates by using Landau–Zener interferences to show the feasibility of achieving quantum two-qubit gates in this system. Our results also demonstrate that the fidelity of two-qubit operations for a QD charge qubit can be comparable to that of spin-based semiconductor qubits^20^,^21^,^22^. For charge qubits, with a sufficiently large coupling energy, the fidelity of two-qubit operations is only limited by the fidelity of the single qubit. Thus, with the reduction of decoherence rate of single qubit using a more sophisticated double QD dispersion^8^, the prospect of semiconductor charge qubits for scalable quantum computation can be considerably improved.

## Results

### Strong inter-qubit coupling

**Figure 1:**
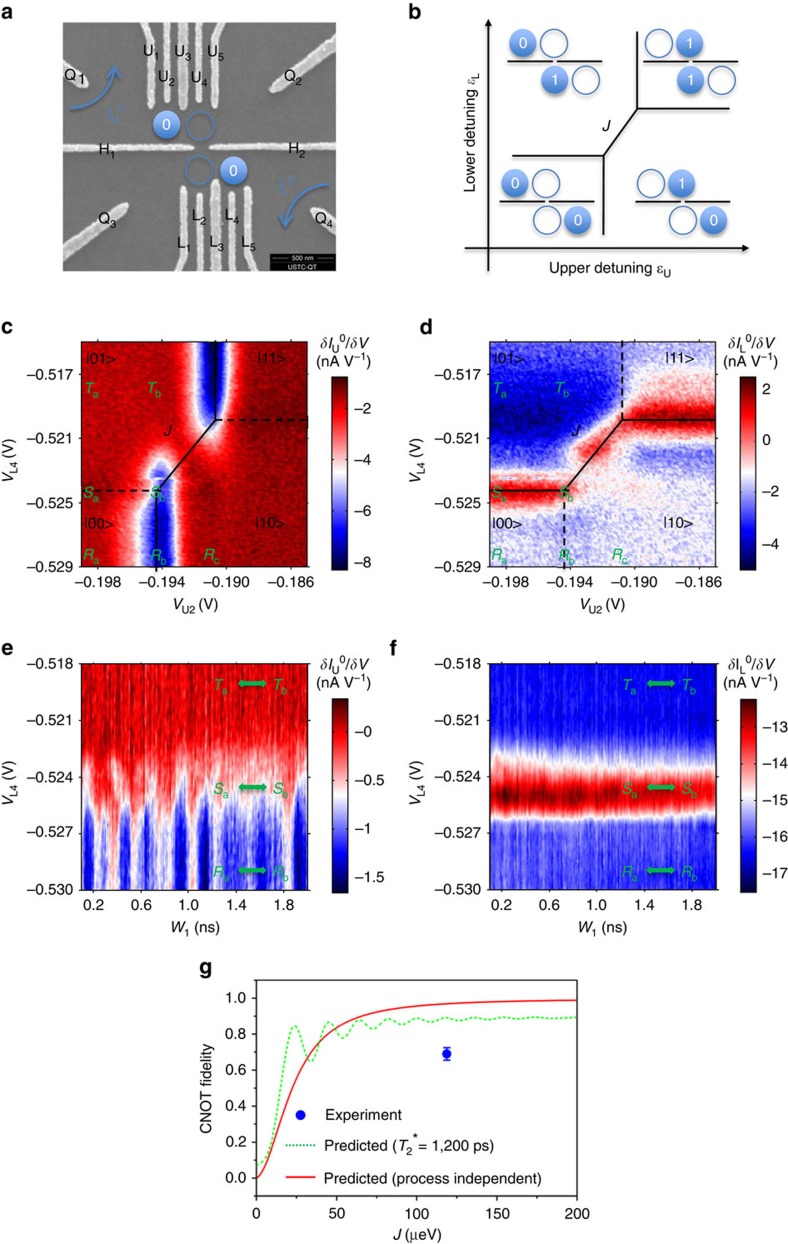
Strong inter-qubit coupling (**a**) Scanning electron micrograph of the device. (**b**) There exists an exchange energy *J* originating from Coulomb repulsion. (**c**,**d**) Experimentally measured *J*. (**e**,**f**) The manipulation of the state of the lower qubit can completely switch the Larmor oscillations of the upper qubit on and off. Points *R*_a_, *S*_a_, and *T*_a_ are the representative working points when the lower qubit is switched from |0> state to |1> state. A pulse is applied on the upper qubit and drives the working point *R*_a_ to *R*_b_, *S*_a_ to *S*_b_, and *T*_a_ to *T*_b_, respectively. (**g**) Red solid curve is the process-independent CNOT gate fidelity as a function of *J*, calculated by solving the master equations. Green curve is the simulated fidelity for 3*π* CNOT gate pulses with *T*_2_*=1.2 ns. The blue dot is our experimentally measured data.

Figure 1a depicts our two-qubit system consisting of two coupled double QD (DQD)s and two quantum point contacts (QPCs). The Hamiltonian of this system is as follows:


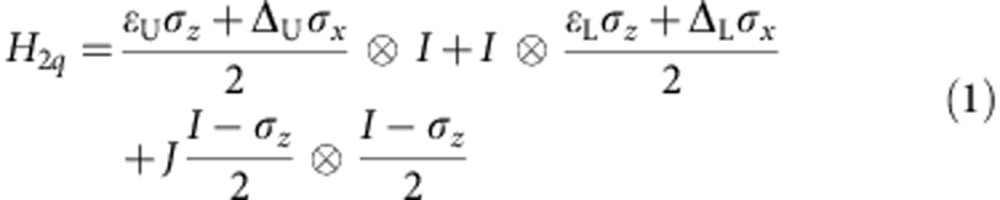


Here, *ɛ*_U_ (*ɛ*_L_) is the energy detuning, Δ_U_=2*t*_U_ (Δ_L_*=*2*t*_L_) is twice the inter-dot tunnelling rate for the upper (lower) DQD, *σ*_*x*_ and *σ*_*z*_ are the Pauli matrixes, *I* is the identity matrix and *J* is the inter-qubit coupling energy. We denote the four eigenstates of the above Hamiltonian by |00>, |10>, |01> and |11>. Normally, the eigenstates are different from the charge states (|RR>, |LR>, |LR> and |LL>) that the QPCs can detect, except when the detuning of each qubit is far away from its balance point (|*ɛ*_U,L_| ≫ 0). We always reset the qubits far from the balance points before applying any gate pulses, where the eigenstates coincide with the charge states. Therefore, in the later contexts, we will describe the evolution of the qubit states in the frame of eigenstates and ignore the unitary transformations between the frame of the eigenstates and that of the charge states.

The inter-qubit coupling energy *J* originates from the Coulomb repulsion between an electron in the upper DQD and another electron in the lower DQD. When the two electrons are closest to each other, the Coulomb interaction energy is higher, by an amount defined as *J*, than it is when the electrons are furthest apart from each other^10^,^11^,^12^,^13^. This is illustrated in Fig. 1b: the abrupt energy shift from state |00> to state |11> is given by *J*. The origin and role of *J* are similar to those of the inter-qubit dipole–dipole interaction between two capacitively coupled semiconductor spin qubits^22^. We will see that a sufficiently large *J* is the key to controlling the coherent rotations of one qubit by manipulating the state of the other qubit, and is therefore the key to realizing two-qubit gates such as CNOT gates.

In our experiment, we are able to achieve very high *J* (*J/h*≈29.0 GHz) compared with other characteristic parameters: Δ_U_≈6.2 GHz and Δ_L_≈6.0 GHz. Figure 1c presents the differential current of the upper QPC and Fig. 1d presents that of the lower QPC. Therefore, Fig. 1c records only the response to the upper detuning, *ɛ*_U_, and Fig. 1d records only the response to the lower detuning, *ɛ*_L_. The two figures together constitute a complete description of Fig. 1b. We find that *J* is equal to ∼119 μeV using the energy–voltage conversion factor obtained from transport measurements of 30 μeV per mV. We can deliberately tune the voltages on the two horizontal gates H_1_ and H_2_ to maximize the inter-qubit coupling energy *J* and simultaneously suppress the direct inter-qubit tunnelling.

Now, we apply a rectangular voltage pulse to one of the upper qubit's gates, U_1_. We initialize both the upper and lower qubits in state |0>, that is, −*ɛ*_U,L_ ≫ *J* ≫ Δ_U,L_>0. Under these conditions, the two qubits are nearly uncorrelated, and only the upper qubit is affected by the voltage pulse. We choose the pulse amplitude such that it will drive the upper qubit exactly to its balance point, that is, from working point *R*_a_ to *R*_b_ as illustrated in Fig. 1c,d. The pulse's combined rise and fall time is measured to be 130 ps on top of the refrigerator. When we sweep the pulse width to longer values, non-adiabatic evolution such as Larmor oscillations is observed. The qubit oscillates between states |0> and |1>, with a probability in each state as a cosine function of the pulse width *W*_1_. In a Bloch sphere, the upper qubit rotates around the *x* axis by an angle proportional to *W*_1_ (refs 4, 5). More information can be found in Supplementary Fig. 1 and Supplementary Note 1.

These are simply regular Larmor oscillations for a single qubit. However, we will observe a difference if we change the state of the lower qubit to |1>, that is, *ɛ*_L_ ≫ *J* ≫ *Δ*_L_>0, while keeping the rest of the system unchanged. When the lower qubit switches from |0> state to |1> state, the upper qubit's balance point shifts toward higher energies by an amount *J*. As a result, the pulse now drives the system from point *T*_a_ to *T*_b_ as illustrated in Fig. 1c,d, meaning the upper qubit is unable to reach its balance point now. We thus expect the Larmor oscillations to disappear.

The experiment clearly demonstrates the above effect. In Fig. 1e,f, we present the Larmor oscillations of the upper qubit conditional on the lower qubit's state. The *x* axis corresponds to the pulse width, *W*_1_. The *y* axis corresponds to the lower qubit detuning, *ɛ*_L_. Figure 1e presents the differential current of the upper qubit and Fig. 1f presents that of the lower qubit. Figure 1f reveals that the lower qubit switches between states as the line *V*_L4_≈−0.525 V is crossed. When *V*_L4_<<−0.525 V, the lower qubit is in the |0> state and Fig. 1e presents the Larmor oscillations of the upper qubit with a frequency equal to Δ_U_=6.2 GHz. When *V*_L4_>>−0.525 V, the lower qubit switches to the |1> state, and in Fig. 1e, it is evident that the Larmor oscillations of the upper qubit disappear. Near the balance point, that is, when −0.526 V<*V*_L4_<−0.524 V, the two qubits should rotate as an entangled state, exhibiting Larmor oscillations at two frequencies, which is irrelevant to our CNOT gate and will be addressed elsewhere.

Figure 1e,f demonstrates that we can completely suppress the upper qubit's Larmor oscillations by switching the lower qubit from the |0> state to the |1> state. A CNOT gate, the logical operation of which is to flip the upper qubit if the lower qubit is in state |0> and to do nothing if the lower qubit is in state |1> can thus obtain its maximum fidelity. We perform theoretical simulations by numerically solving the master equations. Details are provided in Supplementary Fig. 2 and Supplementary Note 2. The simulation successfully reproduces phenomena such as those observed in Fig. 1e,f when the experimentally obtained parameters were used, including *J*=119 μeV. We need to point out that experimentally we present the QPC differential current because of its better signal-to-noise ratio. Our simulation focuses on the state probability, which is directly related to the QPC current. However, the simulation reflects the same features as those of the experimental result.

If we reduce *J*, for instance to 25 μeV, which is the same magnitude as Δ_U_ and Δ_L_, our simulation indicates that in this case, the upper qubit's Larmor oscillations cannot be completely suppressed. There are leakage Larmor oscillations with a 55% amplitude when the lower qubit is switched from state |0> to state |1>. Therefore, a CNOT gate for *J*=25 μeV will achieve a low fidelity. These leakage Larmor oscillations at low J occur because the two balance lines have finite line widths, as shown in Fig. 1c. If *J* is smaller than or comparable to this line width, the two balance lines are smeared out, and the same voltage pulse can drive the upper qubit to its balance point regardless of whether the lower qubit is in state |0> or state |1>. Only if *J* is much larger than this line width will there be no overlap between these two balance lines, and thus no leakage Larmor oscillations. The *J* value required to completely separate the two balance lines is therefore the threshold value for the CNOT gate to achieve maximum fidelity.

We can calculate the dependence of the upper bound of the CNOT gate fidelity on the inter-qubit coupling strength *J* through simulations. Details are provided in the Supplementary Note 2. We present the process-independent fidelity without the dephasing effect as the red solid curve in Fig. 1g. Two important features are apparent: the fidelity increases with increasing *J* and eventually saturates. In our case, *J*=119 μeV, we should, in principle, achieve 97% fidelity for the CNOT gate. However, this estimation is excessively idealistic. It assumes an infinitely long dephasing time, 100% fidelity for the single-qubit gates, and perfect pulse shaping for the two-qubit gates. After considering an inhomogeneous decoherence time 1.2 ns, we simulated the fidelity for CNOT gate operated with 3*π* rotating pulses, shown as the greed dashed curve in Fig. 1g. The fidelity for *J*=119 μeV drops to 0.89. And as we will see, experimentally, we measured the truth table of a CNOT operation and achieved 68% fidelity. Nonetheless, Fig. 1g strongly indicates that *J* is not the major limiting factor in preventing the achievement of perfect fidelity in our experiment. The inter-qubit coupling strength in our device has already been necessarily large to achieve a satisfactory CNOT gate. This is the greatest advancement of this study with respect to earlier experiments.

For simplicity, in this paper, we present the details only for the control of the upper qubit through the manipulations of the lower qubit. Considering the symmetric design, the opposite would certainly be possible, that is, controlling the lower qubit by manipulating the upper qubit.

### Pulse timing

Additional experimental challenges remain in the development of a functional CNOT gate. The voltage pulses required for the implementation of a single-charge qubit gate are already very short (200–500 ps). To demonstrate a CNOT gate, we require up to three sequential ultra-short pulses. These pulses must be carefully synchronized and aligned. Here we demonstrate how we manipulate two pulses, one on the lower qubit and the other on the upper qubit, to coherently rotate the lower qubit and thus control the state of the rotation of the upper qubit. Further details regarding pulse timing are presented in Supplementary Figs 3 and 4, and Supplementary Notes 3 and 4.

**Figure 2:**
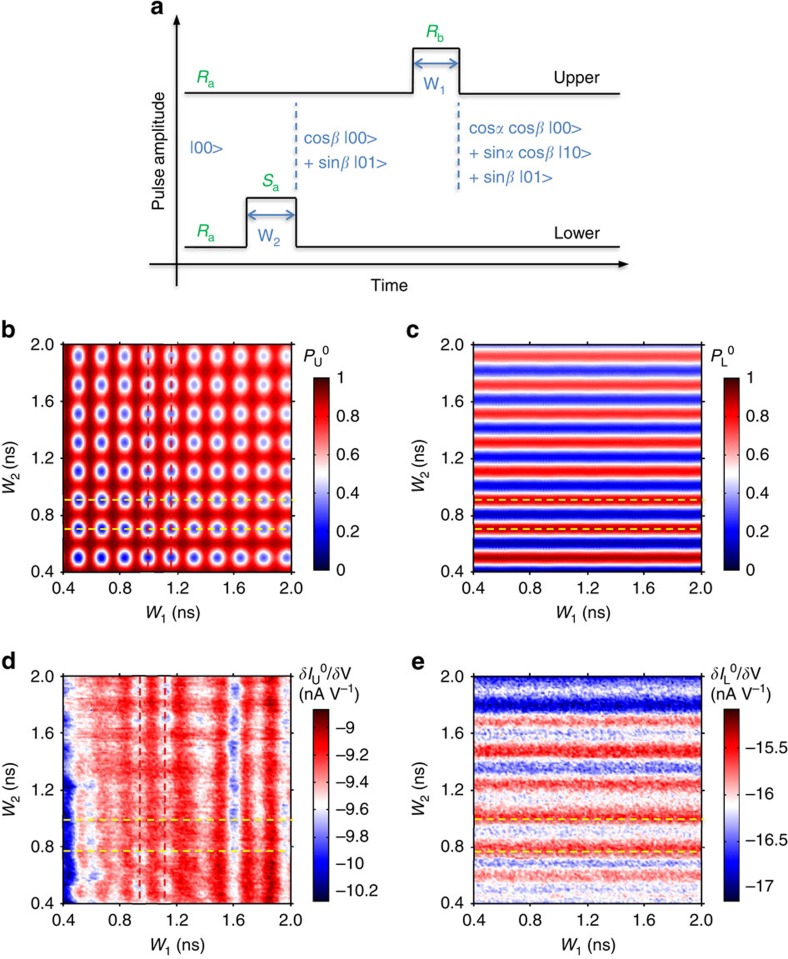
Pulse timing (**a**) Gate pulse flowchart for the coherent control of the upper qubit's Larmor oscillations through pulse driving the lower qubit. The initial working point is at *R*_a_, as specified in Fig. 1c,d. Pulses on the lower and upper qubits drive the working point to *S*_a_ and *R*_b_, respectively. (**b**,**c**) Theoretical simulations of *P*_U_^0^ and *P*_L_^0^, respectively. The red dashed lines indicate two adjacent valleys of the upper qubit's oscillations with respect to *W*_1_. The yellow dashed lines indicate two adjacent valleys in the oscillations of *P*_U_^0^ with respect to *W*_2_, and two adjacent peaks in the oscillations of *P*_L_^0^. (**d**,**e**) Experimentally observed differential current of the upper and lower QPCs, respectively.

A schematic description of the manipulation process is presented in Fig. 2a. Both qubits are initialized in state |0>, that is, working point *R*_a_ as illustrated in Fig. 1b,c. In addition to a rectangular pulse of width *W*_1_ on the upper qubit, as described above, another rectangular pulse of width *W*_2_ is applied to one gate of the lower qubit, L_5_. The lower pulse (*W*_*2*_) first drives the lower qubit to its balance point, that is, from working point *R*_a_ to *S*_a_. After a delay time (∼100 ps) that is much shorter than the dephasing time *T*_2_* (∼1,200 ps) and the relaxing time *T*_1_ (∼19 ns), the upper pulse (*W*_1_) follows and drives the upper qubit to its balance point, that is, from working point *R*_a_ to *R*_b_. If the lower pulse is terminated at 2*nπ*, then the lower qubit will remain in the |0> state. The upper pulse will then rotate the upper qubit by an angle proportional to *W*_1_. By contrast, if the lower pulse is terminated at (2*n*+1)*π*, then the lower qubit will enter the |1> state. Consequently, the upper pulse will have no effect, and the upper qubit will remain in the |0> state regardless of *W*_1_.

Generally, we assume that the pulse of width *W*_2_ rotates the lower qubit by an angle-*β*, and that the pulse of width *W*_1_ rotates the upper qubit by an angle-*α* when the lower qubit is in state |0>. Then, the two qubits will end up in the following entangled state: cos*α* cos*β* |00>+sin*α* cos*β* |10>+sin*β* |01>. The probability of finding the upper qubit in state |0> is *P*_U_^0^=1*−*sin^2^*α* cos^2^*β*, and the probability of finding the lower qubit in state |0> is *P*_L_^0^=cos^2^*β*. Therefore, we predict that *P*_U_^0^ should oscillate with both *W*_1_ and *W*_2_, whereas *P*_L_^0^ should oscillate only with *W*_2_. Moreover, the dependence of *P*_U_^0^ on *W*_2_ is out of phase by *π* compared with *P*_L_^0^. We simulate this process by solving the master equations, as shown in Fig. 2b,c (ref. 16).

Experimentally, we observe the predicted pattern shown in Fig. 2d,e. The QPC differential current for the lower qubit periodically oscillates only along the *W*_2_ axis, whereas that for the upper qubit exhibits oscillations along both the *W*_1_ and *W*_2_ axes. The oscillation frequencies along the *W*_1_ and *W*_2_ axes are Δ_U_ and Δ_L_, respectively. In addition, the dependence on *W*_2_ is out of phase by approximately *π* between the upper and lower qubits, which is as predicted. This finding demonstrates that we can indeed coherently control the Larmor oscillations of the upper qubit.

In addition, the QPC differential current for the lower qubit is invariant with respect to the upper pulse of width *W*_1_. This observation serves as a proof that there is no observable crosstalk between the two qubits.

### CNOT operation truth table

**Figure 3:**
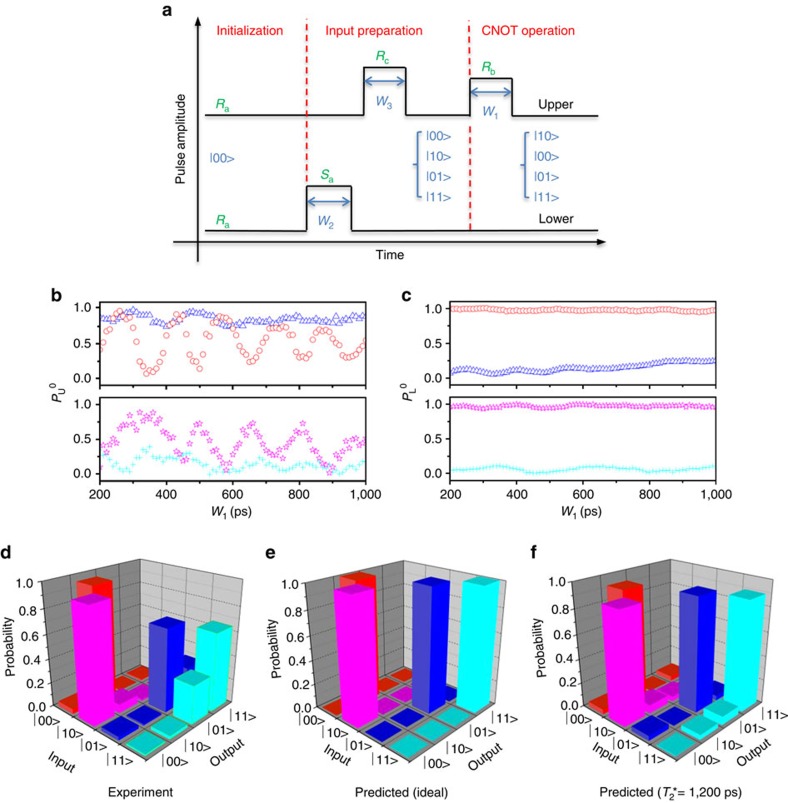
CNOT operation truth table (**a**) Gate pulse flowchart for the CNOT truth table measurements. The initial working point is at *R*_a_, as specified in Fig. 1c,d. *S*_a_, *R*_c_, and *R*_b_ specify the working points that the gate pulses drive the qubits to. (**b**,**c**) Probabilities for the upper and lower qubits, respectively, as functions of the pulse width *W*_1_. For the actual CNOT gate, *W*_1_=360 ps. The red circles, pink stars, blue triangles and green crosses correspond to input states |00>, |10>, |01> and |11>, respectively. (**d**) Experimentally measured probabilities for the CNOT output states. The red, pink, blue and green bars correspond to input states |00>, |10>, |01> and |11>, respectively. (**e**,**f**) Theoretically predicted probabilities: **e** corresponds to no dephasing and **f** corresponds to a dephasing time of 1,200 ps.

Based on the achievement of sufficiently high *J* and proper pulse timing, we now test the CNOT operation and perform truth table measurements to determine its fidelity^8^,^14^,^15^,^16^,^17^,^18^,^19^. Figure 3a presents the process flowchart. In the initialization process, we reset the two qubits to the |00> state, that is, working point *R*_a_. Then, in the input preparation process, we apply certain pulses to both the upper and lower qubits to obtain different input states. By tuning the pulse widths *W*_2_ and *W*_*3*_, we prepare four input states: |00>, |10>, |01> and |11>. Finally, these input states are fed into the CNOT gate, which consists of a *π*-pulse on the upper qubit. The logic of the CNOT gate means that the upper qubit undergoes a *π*-rotation if the lower qubit is in the |0> state and no rotation if the lower qubit is in the |1> state. Therefore, after passing through the CNOT gate, the four input states will be transformed into the |10>, |00>, |01> and |11> states, respectively.

For the first input state |00>, the initial state is directly sent to the CNOT gate without any preparatory pulse. To prepare a |10> input state, a *π* pulse is applied to the upper qubit before the CNOT gate. A *π*-pulse on the lower qubit will yield the |01> input state. Finally, we apply a *π*-pulse to the lower qubit followed by a *π*-pulse with an elevated amplitude on the upper qubit to obtain a |11> input state. A regular *π*-pulse drives the working point from *R*_a_ to *R*_b_ and this pulse drives from *R*_a_ to *R*_c_. The purpose is to force the upper qubit to rotate after the lower qubit has already been switched into the |1> state. Experimentally, because *π*-pulses are too short to be well controlled, we use 3*π*-pulses instead (360 ps for the upper qubit and 390 ps for the lower qubit in our experiment).

The output of the CNOT gate for each of the four input states is read through the QPC current. We use the pulse-modulation technique developed in previous studies to convert the QPC current into a state probability^8^. Further details are provided in Supplementary Fig. 1 and Supplementary Note 1. In Fig. 3b,c, we sweep the pulse width of the CNOT gate (*W*_1_) and measure the probabilities *P*_U_^0^ and *P*_L_^0^ after generating each of the four input states. As expected, *P*_U_^0^ exhibits Larmor oscillations for input |00>. For input |10>, the Larmor oscillations are shifted by a phase of *π*. For inputs |01> and |11>, *P*_U_^0^ exhibits essentially no oscillation because the lower qubit has been switched into state |1>. The difference between the two inputs is that *P*_U_^0^ remains at a high level for input |01> and at a low level for input |11>. *P*_L_^0^ exhibits essentially no dependence on *W*_1_ because the upper pulse does not affect the lower qubit.

From Fig. 3b,c, we extract the values of *P*_U_^0^ and *P*_L_^0^ at *W*_1_=360 ps, which corresponds to a 3*π*-pulse on the upper qubit and therefore a CNOT gate. Based on these values, we obtain the truth table for the CNOT gate, as illustrated in Fig. 3d. For comparison, we simulated the truth table for an ideal CNOT gate and for a CNOT gate with an inhomogeneous dephasing time of 1,200 ps, as shown in Fig. 3e,f, respectively. The detailed values of these truth tables are provided below (ref. 15):


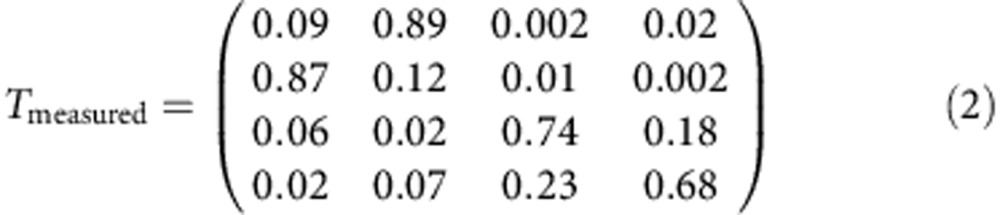



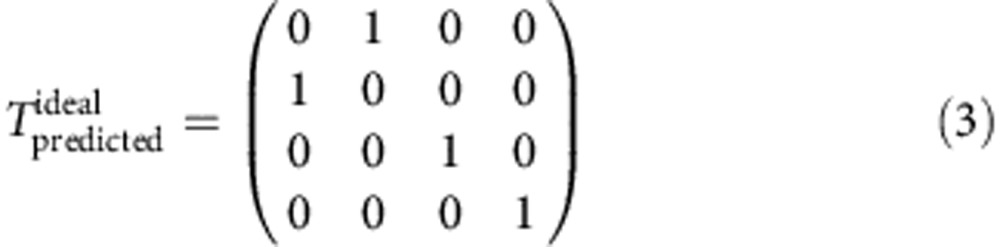



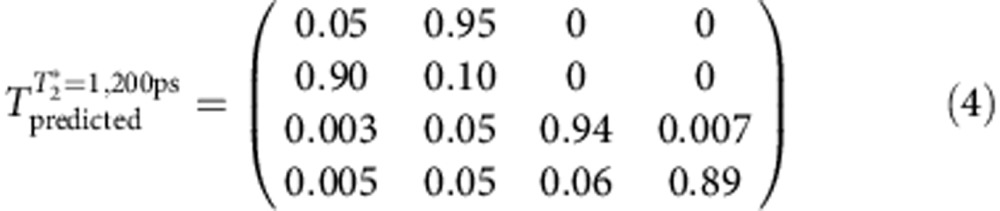


The measured fidelity is 0.68. We must reiterate that the inter-qubit coupling strength *J* is not the limiting factor responsible for this imperfect fidelity because *J* is already sufficiently large to allow us to completely switch the rotations of the upper qubit on and off by manipulating the state of the lower qubit. We believe that there are two main factors that account for the deviation of the measured fidelity from 1. First, the relatively short dephasing time of a single-charge qubit causes errors in single-qubit operations, and these errors are carried over into the two-qubit operations. Once the lower qubit suffers dephasing, the lower qubit state will contain a |0> component even when it should be in the |1> state. This leakage to the |0> state will cause the suppression of the upper qubit's Larmor oscillations to be incomplete. In combination with the dephasing of the upper qubit, this effect will degrade the ultimate CNOT gate fidelity. By comparing Fig. 3e,f, our simulation reveals that the predicted CNOT gate fidelity decreases to 89% when a qubit dephasing time of 1,200 ps is considered^5^.

Second, the pulse shaping of multiple ultra-short pulses is extremely challenging and cannot be made ideal. The relatively short dephasing time in our system requires us to complete gate operations as quickly as possible. Although this forces us to boost the gate operation speed, which proves to be helpful, it also increases the risk of poor pulse shaping. In particular, we observe that the finite nature of the pulse rising and falling times makes the precise tuning of the pulse width difficult. In addition, we observe that the increase in the pulse amplitude with increasing pulse width makes it difficult to accurately control the amplitude of each pulse and to align the amplitudes of multiple pulses. The accumulation of all these errors gives rise to deviation between the final output qubit state and the desired qubit state. This might be the primary reason for the CNOT fidelity to drop to ∼68%.

Although this fidelity does not contain the part of phase rotation, its value is almost as high as that of spin-based semiconductor qubits. The relatively short dephasing time of charge qubits has always been an obstacle preventing the serious consideration of the possibility of multiple-charge qubits. However, the large intra-qubit coupling and inter-qubit coupling originating from the direct Coulomb interaction between electron charges enable us to operate the CNOT gate at a very high clock speed (a few GHz) and to maintain the gate fidelity at a satisfactory level. Coulomb interactions are significant in various types of multi-qubit systems. For example, spin qubits in silicon (Si)-based QDs have recently demonstrated remarkably long coherence times^23^,^24^,^25^,^26^. High-quality single-electron spin gates have been demonstrated using Si. However, long-range inter-qubit coupling still originates from the Coulomb interaction. Two-qubit gates using Si still suffer from high charge noise and therefore still require further development. We hope that our demonstration of two-qubit gates based on the Coulomb interaction may offer inspiration for the investigation of semiconductor multi-qubits.

There is still a room to improve the fidelity of our electron charge two-qubit gates. The errors originating from imperfect pulse shaping are deterministic and could be corrected with further progress in high-frequency technology. We hope that the advancement of picosecond pulse generators and the incorporation of on-chip transmission lines will help us to improve the fidelity of our single- and double-qubit gates. Moreover, the qubit dephasing effect is intrinsic, and new materials or architectures will be necessary to achieve significant improvements. Recent progress in the field of hybrid qubits has demonstrated that fast operating speeds and long coherence times can be simultaneously achieved in electron charge qubits by engineering an energy structure with certain excited states^8^. If similar schemes can be applied to increase *T*_2_* in our system by perhaps 1 to 2 orders of magnitude, the fidelity of the two-qubit CNOT gate could markedly increase.

### Prospective quantum logic gates

In the CNOT truth table measurement, we considered only the amplitudes of the quantum states. However, Fig. 2 previously illustrated the quantum nature of the CNOT gate, which has no counterpart among classical gates. The amplitudes of the quantum states of both the upper and lower qubits can be set to any arbitrary superposition value, corresponding to rotations by arbitrary angles around the *x* axis in each Bloch sphere.

**Figure 4:**
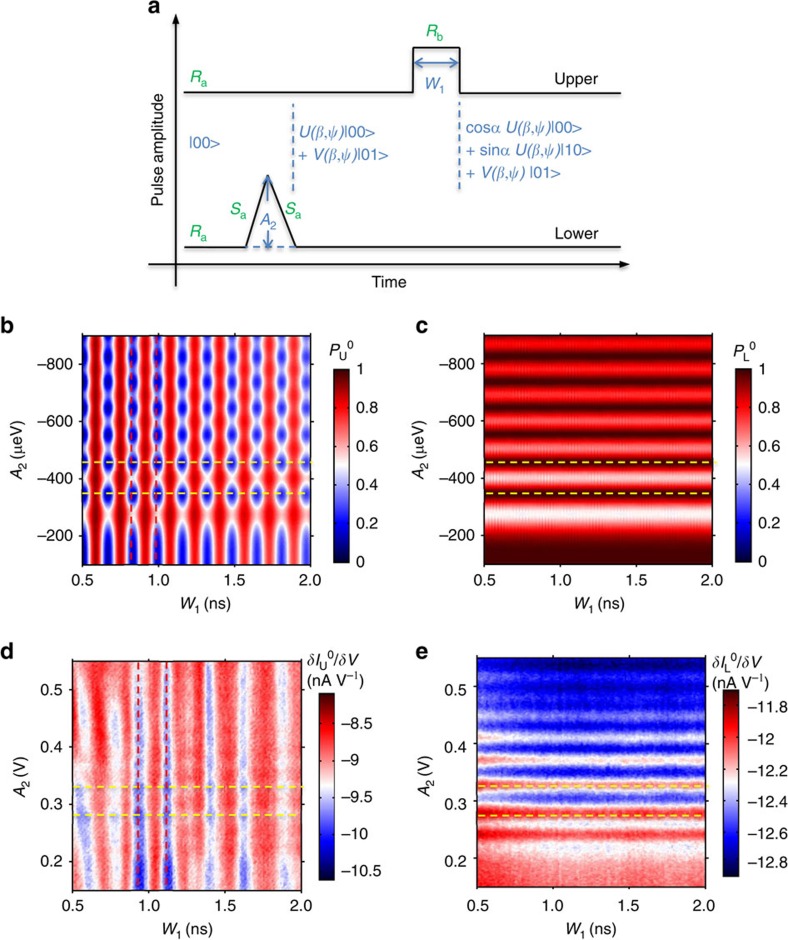
Prospective quantum logic gates (**a**) Gate pulse flowchart for the use of the lower qubit's phase to control the upper qubit's Larmor oscillations. The initial working point is at *R*_a_, as specified in Fig. 1c,d. Triangular pulse on the lower qubit drives the working point through *S*_a_ when the pulse amplitude is large enough. Upper pulse drives the working point to *R*_b_. (**b**,**c**) Theoretical simulations of *P*_U_^0^ and *P*_L_^0^ in which we sweep the lower qubit's pulse amplitude to control the Larmor rotations of the upper qubit. The red dashed lines indicate two adjacent valleys of the oscillations of the upper qubit with respect to *W*_1_. The yellow dashed lines indicate the response to *A*_2_, which are two valleys for *P*_U_^0^, whereas two peaks for *P*_L_^0^. (**d**,**e**) Experimental results for the differential current of the upper and lower QPCs, respectively.

Furthermore, we will show that we can vary both the phase and amplitude of the qubit's states while preserving the CNOT logic. As illustrated in Fig. 4a, a pulse with a fixed width of 100 ps is applied to the lower qubit. This pulse width is set to be shorter than the rise and fall times combined, and therefore, the pulses can be regarded as triangular. This pulse width is also small compared with *T*_2_* away from the balance point, which was estimated to be ∼300 ps by studying the inhomogeneous broadening of the photon-assisted tunnelling line width^5^. We initialize the lower qubit in state |0> and sweep the lower pulse amplitude. The lower qubit is driven to pass through its balance point if the pulse amplitude is larger than its detuning, that is, from working point *R*_a_ to cross *S*_a_. This adiabatic passage through the balance point induces the Landau–Zener–Stuckelberg effect, corresponding to rotation around both the *x*- and *z* axis in the Bloch sphere^6^.

Immediately following the lower pulse, a rectangular pulse is applied to the upper qubit and induces Larmor oscillations in the upper qubit, that is, from working point *R*_a_ to cross *R*_b_. Here we demonstrate that the Larmor oscillations of the upper qubit are controlled by the lower qubit's phase accumulation caused by Landau–Zener–Stuckelberg interference. First, let us suppose that the triangular pulse can independently drive the lower qubit from the |0> state into the *U(β,ψ)* |0>+*V(β,ψ)* |1> state, where *U*^2^*(β,ψ)*+*V*^2^*(β,ψ)*=1, and that the rectangular pulse can independently drive the upper qubit from the |0> state into the cos*α* |0>+sin*α* |1> state if the two qubits are completely uncorrelated.

In reality, the two qubits are coupled, and the CNOT gate logic ensures that the upper triangular pulse can only cause the upper qubit to rotate when the lower qubit is in the |0> state. Consequently, after both the lower and upper pulses, the final entangled two-qubit state will be as follows: *U(β,ψ)* cos*α* |00>+*U(β,ψ)* sin*α* |10>+*V(β,ψ)* |01>. The probability of finding the upper qubit in state |0> is *P*_U_^0^=1-*U*^2^*(β,ψ)* sin^*2*^*α*, and the probability of finding the lower qubit in state |0> is *P*_L_^0^=*U*^2^*(β,ψ)*. *U*^2^*(β,ψ)* oscillates with both the amplitude (through *β*) and phase (through *ψ*) of the lower qubit's state. However, the oscillation of the phase is much faster than that of the amplitude. Therefore, in the time window of our experiment, we predominantly observe periodic oscillations with the phase. Thus, *P*_U_^0^ will exhibit cosine oscillations with both *W*_1_ (through *α*) and *A*_2_ (mainly through *ψ*), and *P*_L_^0^ will oscillate only with *A*_*2*_(predominantly through *ψ*). Again, we note that the dependence of *P*_U_^0^ on *A*_2_ is out of phase by *π* compared with that of *P*_L_^0^. In Fig. 4b,c, we present the simulated responses of *P*_U_^0^ to *A*_2_, respectively.

This interpretation explains the data depicted in Fig. 4d,e, where we present the oscillations of the upper qubit with *W*_1_ and *A*_2_. As expected, the upper qubit exhibits not only Larmor oscillations with respect to *W*_1_ through angle-*α* but also oscillations with *A*_2_ through the phase *ψ.* Its dependence on *ψ* is also out of phase by *∼π* as compared with that of the lower qubit. This indicates that the phase of the lower qubit's state can be used to control the state of the upper qubit. Our CNOT gate is thus proven to operate on quantum states of the qubits. In Supplementary Fig. 3 and Supplementary Note 3, we demonstrate that we can even rotate the phase and amplitude of both the upper and lower qubits while preserving the CNOT gate quantum logic.

We also need to point out that further experiment is still needed to explicitly quantify the degree of entanglement between the two qubits during the operation process to realize quantum two-qubit logic gates.

## Discussion

In summary, a string of technical accomplishments, including the achievement of a strong inter-qubit coupling and the synchronization of multiple ultrafast-pulses, enabled us to demonstrate universal two-qubit operations in an all electrically controlled semiconductor charge system. CNOT gate truth table shows high fidelities comparable to that of electron spin two-qubit gates. However, a quantum process tomography measurement^22^ is required to definitively compare the qubit metrics of the two systems. At current stage, trading shorter dephasing time for faster qubit operation time, charge qubits can perform well in the two-qubit level. Optimistically, we argue that with a better control of pulse shape and a better design of the dispersion relations, which are completely deterministic, the semiconductor charge qubits may become a force to contend with in the scalable quantum computation arena.

## Methods

### Devices

The two-DQD device was defined via electron beam lithography on a molecular beam epitaxially grown GaAs/AlGaAs heterostructure. A two-dimensional electron gas is present 95 nm below the surface. The two-dimensional electron gas has a density of 3.2 × 10^11^ cm^−2^ and a mobility of 1.5 × 10^5^ cm^−2^V^−1^s^−1^. Figure 1a presents a scanning electron micrograph of the surface gates. Five upper gates U_1_–U_5_ and two horizontal gates H_1_ and H_2_ form the upper DQD. Five lower gates L_1_–L_5_ and two horizontal gates H_1_ and H_2_ form the lower DQD. The horizontal gates H_1_ and H_2_ also tune the capacitive coupling strength between the upper and lower DQDs. Direct electron tunnelling is suppressed between the two DQDs by ensuring adequate negative bias voltages on gates H_1_ and H_2_. The four gates Q_1_–Q_4_ define QPCs for the monitoring of the charge status on each DQD.

### Measurements

The experiments are performed in an Oxford Triton dilution refrigerator with a base temperature of 10 mK. Two Agilent 81134A pulse generators, which have a rise time of 65 ps and a time resolution of 1 ps, are used to deliver fast pulse trains through semi-rigid coaxial transmission lines to the device. Standard lock-in modulation and detection techniques are used for the charge-sensing read-out. Through electronic transport and photon-assisted tunnelling measurements, wherein the electron energy can be read directly from the source-drain bias voltage and the photon frequency, we conclude that the energy–voltage lever arm is ∼100 μeV per mV for the barrier gates (U_1_, U_5_, L_1_ and L_5_) and 30 μeV per mV for the plunger gates (U_2_, U_4_, L_2_ and L_4_).

## Additional information

**How to cite this article:** Li, H. O. *et al*. Conditional rotation of two strongly coupled semiconductor charge qubits. *Nat. Commun.* 6:7681 doi: 10.1038/ncomms8681 (2015).

## Supplementary Material

Supplementary Information Supplementary Figures 1-4, Supplementary Notes 1-4 and Supplementary References
