# Supplementary material for: Conditional rotation of two strongly coupled semiconductor charge qubits
Source: Nat Commun. 2015 Jul 17;6:7681. doi: 10.1038/ncomms8681 (PMC4518268; doi:10.1038/ncomms8681)
Supplement: Supplementary Information — Supplementary Figures 1-4, Supplementary Notes 1-4 and Supplementary References [file ncomms8681-s1.pdf]

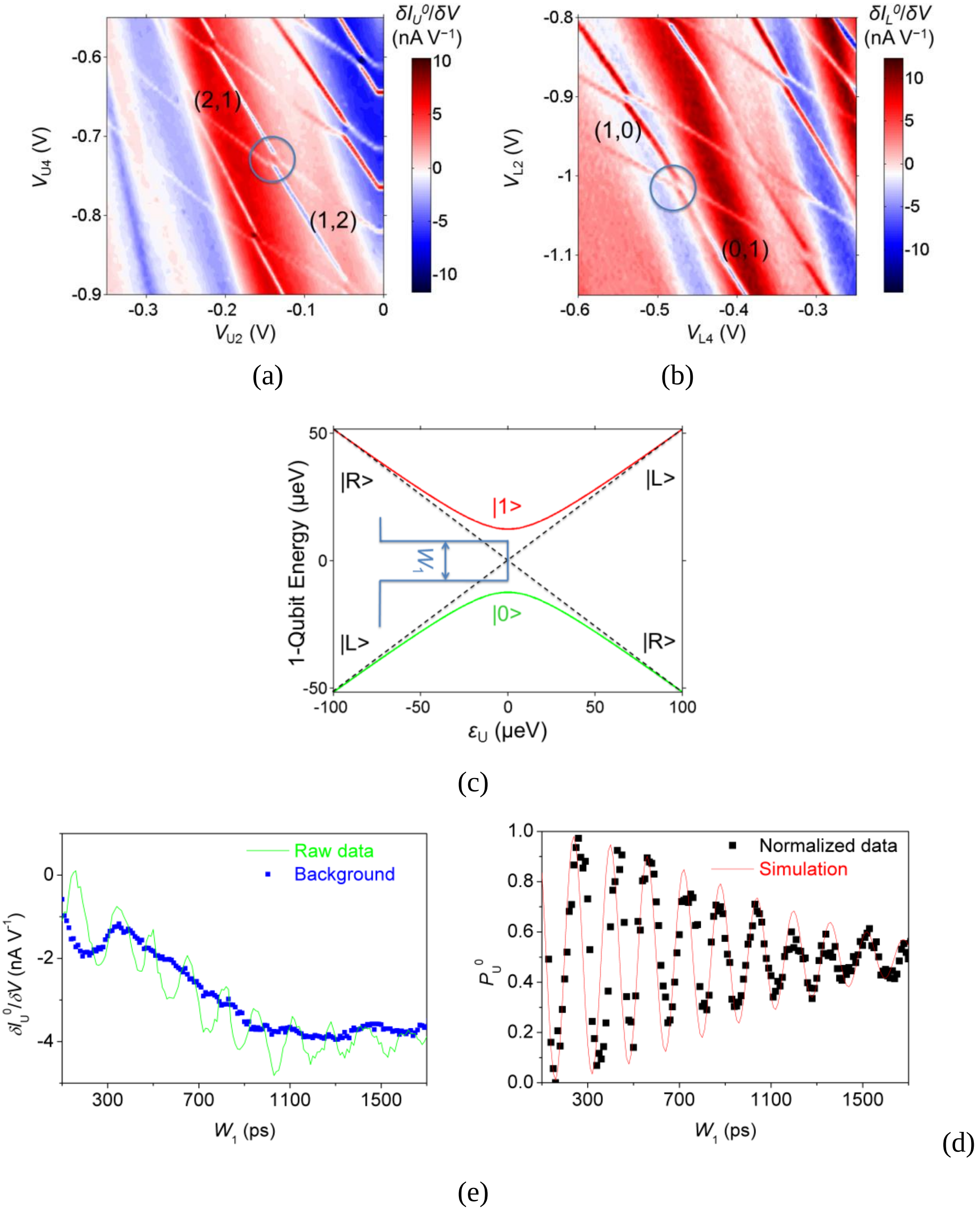


**Supplementary Figure 1 Single-qubit Manipulation and QPC Measurement (a) and (b)** Phase diagrams for the upper and lower DQDs, respectively. **(c)** Calculated energy levels of the upper qubit when the lower qubit is fixed at |0> state. A rectangular pulse is applied to the upper qubit to drive it from the |0> state to its balance point and induce Larmor oscillations. **(d)** The green solid line represents the raw data of the Larmor oscillations of the upper qubit. The blue dotted line represents the background curve. **(e)** The black dotted curve represents normalized Larmor oscillations obtained by dividing the raw data by the background. The red solid line represents the theoretical fit.

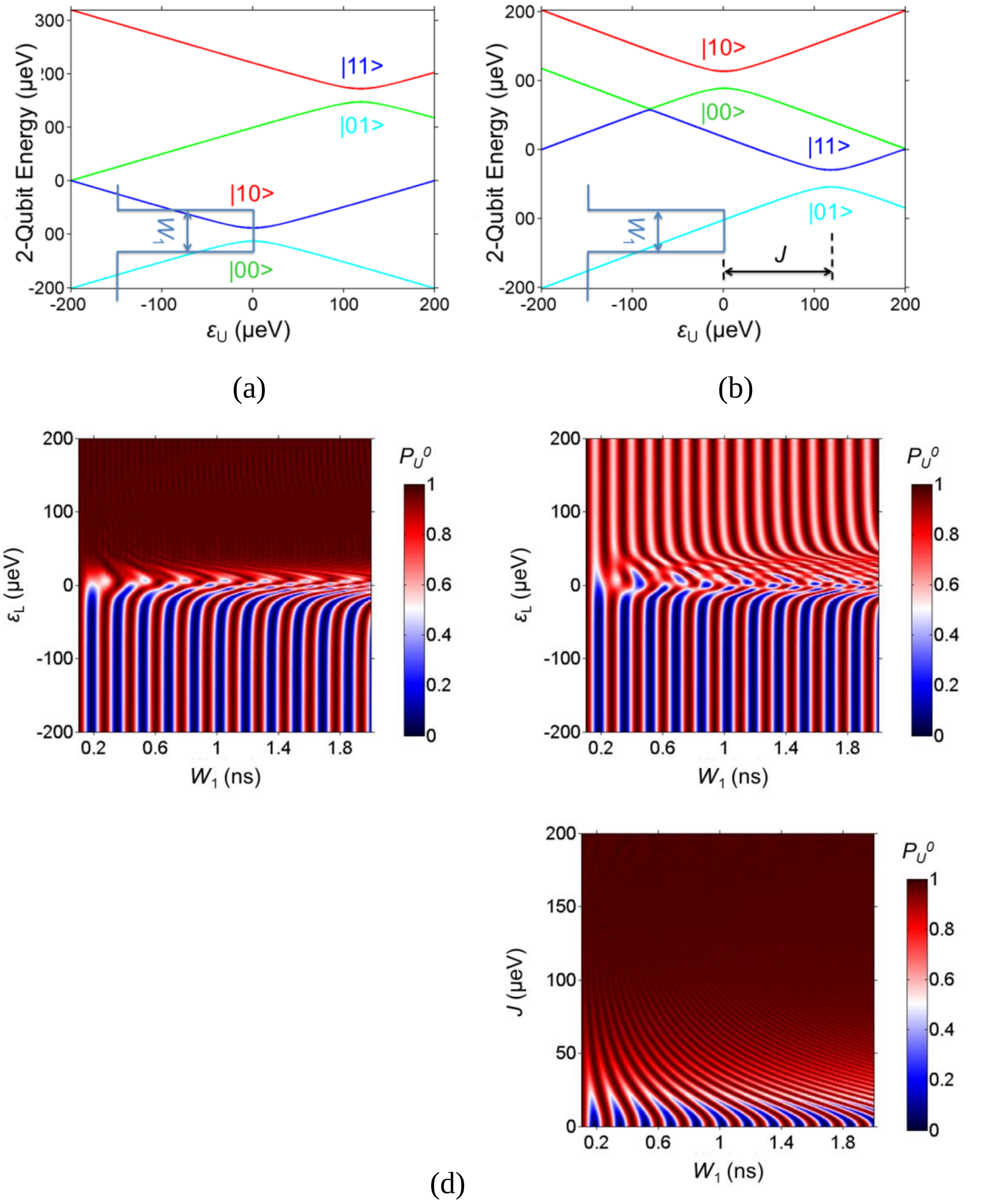


(a) (b)

(c) (d)

(e)

**Supplementary Figure 2 Simulation through Solving the Master Equations (a) and (b)** For $J$ = 119 μeV, the calculated two-qubit energy levels when the lower qubit is set at |0> and |1> state, respectively. The same pulse that can drive the upper qubit from |0> state to its balance point in case (a) cannot drive to the balance point any more in case (b), due to the energy shift caused by a strong two-qubit coupling energy $J$. **(c)**The simulated response ofthe upper qubit to the upper pulse width, under varying values of the lower qubit detuning for $J$ = 119 μeV. **(d)**Same simulations for $J$ = 25 μeV.**(e)** The dependence on $J$ of the leakage Larmor oscillations of the upper

qubit when the lower qubit is fixed at the |1> state ($\varepsilon_L$ = 200 μeV).

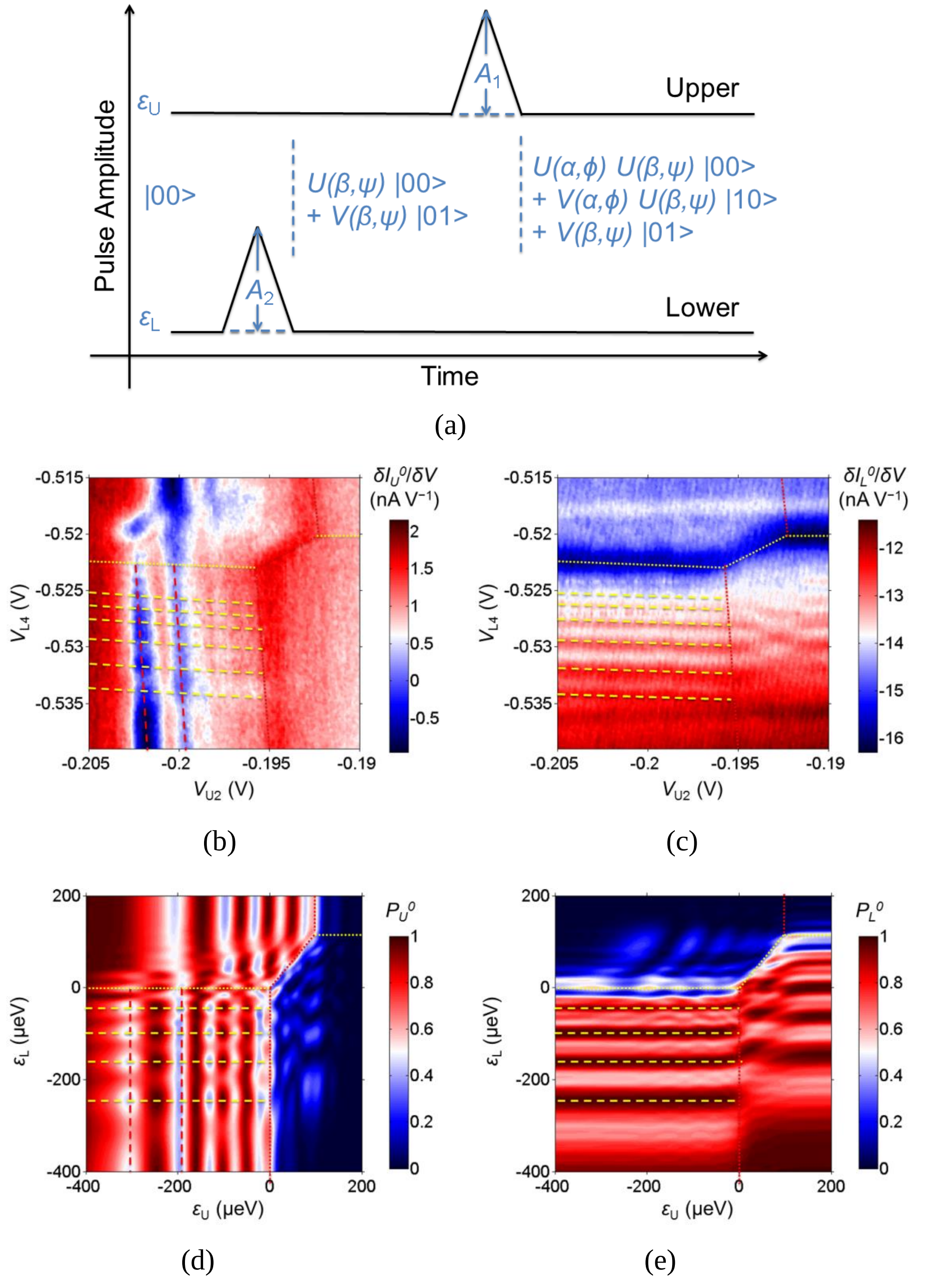


**Supplementary Figure 3 Controlled-Universal-Rotations (a)** Gate pulse flowchart for universal single-qubit manipulation performed in combination with the CNOT gate. **(b) and (c)** Experimental results for the differential currents of the upper and lower QPCs, respectively. The red dashed lines indicate two adjacent valleys of the oscillations of the upper qubit with respect to *the detuning of the upper qubit*. The yellow dashed lines indicate a few adjacent valleys in the upper qubit's response to the detuning of the lower qubit, whereas indicate adjacent peaks in the lower qubit's response to the detuning of the lower qubit. The red and yellow dotted lines indicate

the detuning balance lines. **(d) and (e)** Theoretical simulations of $P_U^0$ and $P_L^0$.

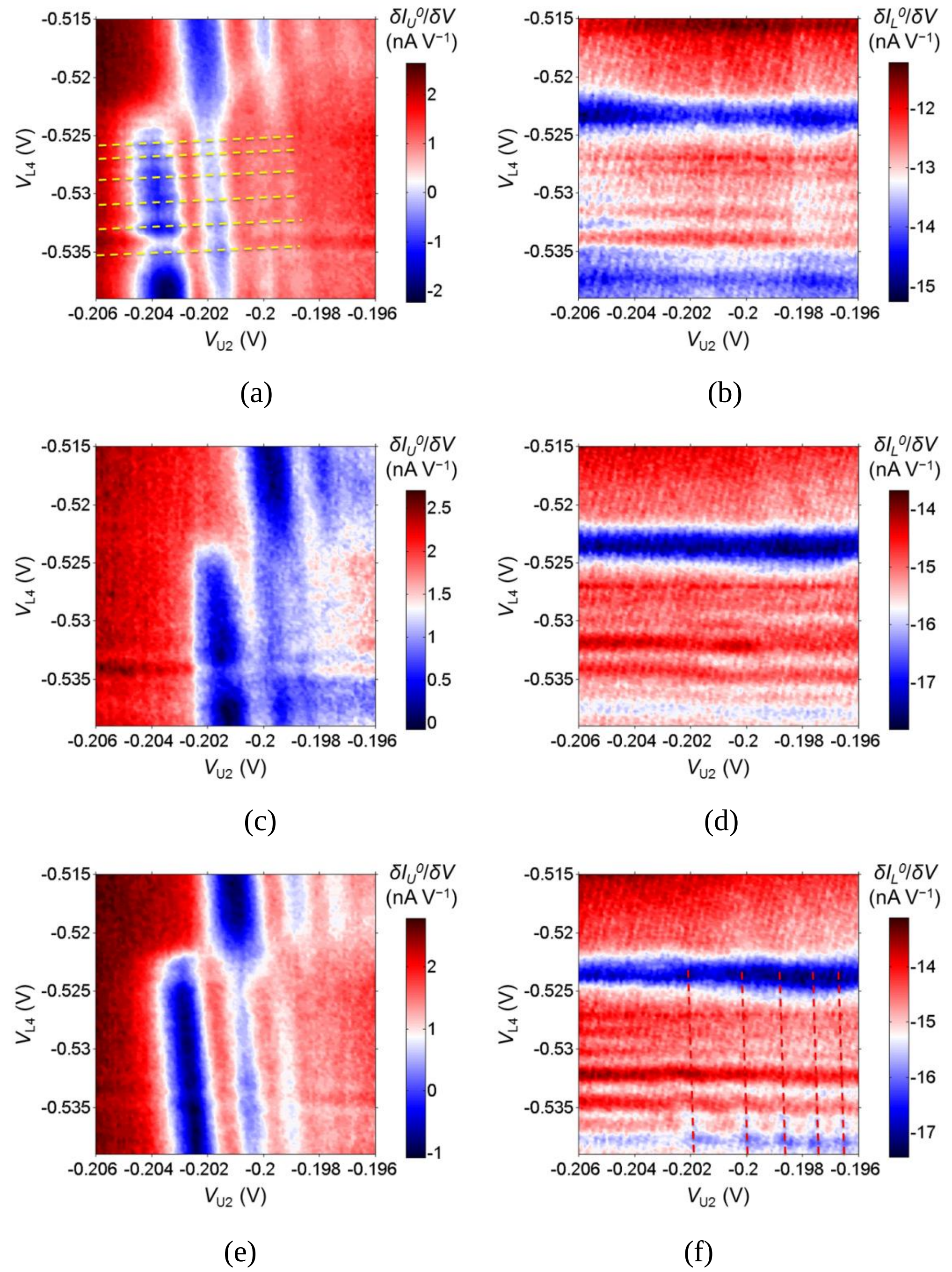


**Supplementary Figure 4 Pulse Synchronization (a) and (b)** Differential current of upper and lower QPCs, respectively, when the predetermined delay time from the end of pulse $W_2$ to the beginning of pulse $W_1$ is set as -100 ps at the pulse generator. The upper qubit is controlled by the lower qubit, meaning that the upper pulse lags behind the lower pulse. **(c) and (d)** The predetermined delay time is -200 ps. The two pulses are most likely synchronized in this case. **(e) and (f)** The predetermined delay time is -300 ps. The lower qubit is controlled by the upper qubit, meaning that the upper pulse is ahead of the lower pulse.

**Supplementary Note 1 Single-qubit Manipulation and QPC Measurement**

We independently consider the charge energy diagrams for the upper and lower DQDs, as shown in Supplementary Figures 1(a)-(b), respectively. The electron charge occupation on either side of the upper DQD is tuned by two upper plunger gates, $U_2$ and $U_4$, and detected by the upper QPC. In the same way, the charge occupation on either side of the lower DQD is tuned by two lower plunger gates, $L_2$ and $L_4$, and detected by the lower QPC.

We operate the two-qubit system between charge occupations of (2,1) and (1,2) for the upper DQD and between charge occupations of (1,0) and (0,1) for the lower DQD, as indicated by the circles in Supplementary Figures 1(a)-(b). The upper energy detuning $\varepsilon_U$, obtained through the voltage detuning between $U_2$ and $U_4$, controls the (2,1) $\leftrightarrow$ (1,2) transition for the upper DQD. Similarly, the lower energy detuning $\varepsilon_L$, obtained through the voltage detuning between $L_2$ and $L_4$, controls the (1,0) $\leftrightarrow$ (0,1) transition for the lower DQD.

Generally, we measure the differential current of the QPC because of its better signal-to-noise ratio. In two-qubit gate operations, we need to know the state probability explicitly to measure the fidelity. For this purpose, we modify the QPC measurement technique [1]. The pulse voltage on gate $U_1$, which is used to induce coherent oscillations, is modulated by a 200 Hz square wave. The same 200 HZ square wave also triggers the lock-in that is used to measure the modulated QPC current. Therefore, the ratio between the lock-in-measured oscillation amplitude and the largest oscillation amplitude tells us the state probability if we regard the largest oscillation amplitude as having a probability 1.

In Supplementary Figure 1(c), we calculated energy levels of the upper qubit when the lower qubit is fixed at |0> state. When the upper qubit is initialized at |0> state, we could apply a rectangular pulse towards the positive detuning direction to the balance point and induce Larmor oscllaitons. The green solid line in Supplementary Figure 1(d) represents a coherent oscillation curve of the upper qubit. Here, the upper detuning is tuned such that the upper pulse drives it exactly to its balance point. We can see that there is an overall shift in background with pulse width. Different pulse widths result in different average voltages on gate $U_1$. Because the QPC is operated approximately within its most sensitive range, which is not strictly linear, a shift in $U_1$ results in a shift in the sensitivity of the QPC current. This is most likely the origin of the background shift with respect to the pulse width. We can eliminate this shifting background by dividing the QPC signal by a pure background, which can be obtained at lower detuning value where Larmor oscillations do not occur. The blue dotted line in Supplementary Figure 1(d) shows such a background curve.By dividing the raw Larmor oscillation by the background, we can obtain the normalized state probability for Larmor oscillation, as indicated by the black dotted curve in Supplementary Figure 1(e). This curve can be well fitted with a decaying cosine oscillation: $a_0\, exp(-(W_1/T_2^*)^2\, cos(2\pi\Delta_U W_1 + b_0) + a_1 W_1 + a_2$. Through fitting, we obtain $T_2^* = 1200$ ps, $\Delta_U = 6.2$ GHz, $a_0 = 0.50$, $a_1 = 0$, $a_2 = 0.50$, and $b_2 = 0.03\pi$.

**Supplementary Note 2 Simulation through Solving the Master Equations**

The Hamiltonian described in equation (1) can be written in matrix form as follows:

$$H_{2q} = \frac{1}{2}\begin{vmatrix} \varepsilon_U + \varepsilon_L & \Delta_U & \Delta_L & 0 \\ \Delta_U & -\varepsilon_U + \varepsilon_L & 0 & \Delta_L \\ \Delta_L & 0 & \varepsilon_U - \varepsilon_L & \Delta_U \\ 0 & \Delta_L & \Delta_U & -\varepsilon_U - \varepsilon_L + 2J \end{vmatrix} \qquad (1)$$

The evolution of the two-qubit system can be described by a time-dependent 4x4 density matrix $\rho$, which obeys the master equations:

$$\frac{d\rho}{dt} = -\frac{i}{\hbar}[H_{2q}, \rho] \qquad (2)$$

To numerically solve these equations, we must first separate the real and imaginary parts of the density matrix. Let us define $W$ = *real(ρ)* + *imaginary(ρ)*. $W$ is a real matrix and *trace(W)* = 1. Because both $\rho$ and $H_{2q}$ are Hermitian and $H_{2q}$ is real, we can transform the master equations in terms of $\rho$ into those in terms of $W$:

$$\frac{dW}{dt} = -\frac{i}{\hbar}[H_{2q}, W^T] \qquad (3)$$

Here $W^T$ is the transpose of $W$. The transformed master equations consist of 15 correlated real differential equations and can be numerically solved using popular scientific programming languages such as MATLAB or Python. The diagonal elements of $W$ are identical to those of $\rho$ and contain all the information that we need:

$$P_U^0 = \rho_{1,1} + \rho_{3,3} = W_{1,1} + W_{3,3} \qquad (4)$$

$$P_L^0 = \rho_{1,1} + \rho_{2,2} = W_{1,1} + W_{2,2} \qquad (5)$$

The initial conditions for $W$ and $\rho$ will also be the same if we initialize $\rho$ to be real. For two-qubit operations, we always begin with conditions such that $|\varepsilon_{U,L}| >> 0$ for the purpose of initializing the two-qubit system in the |00> state. Considering the thermal activation at finite temperature, the initial conditions should be as follows:

$$W(t = 0) = \rho(t = 0) = \frac{\mathrm{diagonal}\,(e^{-E/kT})}{\mathrm{trace}\,(\mathrm{diagonal}\,(e^{-E/kT}))} \qquad (6)$$

Here, $E$ is a matrix whose i[th] diagonal element is the i[th] eigenvalue of $H_{2q}$. The relaxation and dephasing mechanisms can also be incorporated:

$$\frac{dW}{dt} = -\frac{i}{\hbar}[H_{2q}, W^T] - \Gamma_1(W - W(t = 0)) - \Gamma_2 W \qquad (7)$$

$\Gamma_1$ is the relaxation rate matrix. $\Gamma_2$ is the dephasing rate matrix. Because we can measure the relaxation rate by varying the sampling frequency of the operating gate pulses and then numerically compensate for the relaxation effect, we ignore $\Gamma_1$ in our simulation. The dephasing rate gives rise to decaying probabilities and reduced gate fidelities. Through comparison with the experiment, we conclude that the inhomogeneous dephasing time $T_2^* = 1/\Gamma_2$ at the balance point should be approximately 1200 ps, which is of the same magnitude as the dephasing times in previous experiments [2].

Using the initial conditions in Supplementary Equation 6, we numerically integrate the Supplementary Equation 7 over time, thereby solving for the matrix elements of *W* at any given time. Then, using Supplementary Equations 4 and 5, we complete the simulation of this two-qubit system. By performing parallel computation using a graphical processing unit (GPU), we can tremendously increase the speed of our simulation. Furthermore, our method can be conveniently extended to multi-qubit systems.

As an example, we demonstrate how we simulate the expected CNOT fidelity. First, we evaluate the CNOT fidelity in a simple manner. The essence of a CNOT gate lies in the complete on/off switching of the upper qubit's Larmor oscillations through the manipulation of the state of the lower qubit. If the dephasing issue is neglected, the Larmor oscillation amplitude of the upper qubit should decrease from 1 to 0 when the lower qubit switches from state |0> to state |1>. In Supplementary Figures 2(a)-(b), for $J$ = 119 μeV we calculated the two-qubit energy levels when the lower qubit is set at |0> and |1> state, respectively. The same pulse that can drive the upper qubit from |0> state to its balance point in the former case cannot drive it to the balance point any more, due to the energy shift cause by a strong two-qubit coupling energy $J$. This behavior is observed in our experiment, as shown in Figs. 1(e)-(f). In Supplementary Figures 2(c), we simulate the response of the upper qubit to the pulse width for $J$ = 119 μeV. The Larmor oscillations of the upper qubit achieve a magnitude of 0.97 when the lower qubit is in the |0> state ($\varepsilon_L << 0$) and drop to 0 when the lower qubit is in the |1> state ($\varepsilon_L >> 0$).

However, the Larmor oscillations will not be completely suppressed if $J$ is not sufficiently large. This is because the balance point of the upper qubit exhibits a very small shift so that the upper gate pulse still has a chance to reach it. In Supplementary Figures 2(d), we simulate the case of $J$ = 25 μeV. There are obvious leakage Larmor oscillations when the lower qubit is in the |1> state. By varying $J$, we simulate these leakage Larmor oscillations with respect to the pulse width $W_1$. In the simulation, $\varepsilon_L$ is fixed to 200 μeV to ensure that the lower qubit is in the |1> state. Supplementary Figure 2(e) clearly shows that the leakage oscillations become weaker as $J$ increases and will be suppressed when $J$ is sufficiently large.

The leakage oscillations represent the leakage probability that the upper qubit will flip even if the lower qubit is set to state |1>. Let $A_k(J)$ be this leakage probability; then, we can define the CNOT fidelity as $F(J) = 1 - A_k(J)$. From Supplementary Figure 2(e), for any given value of $J$, we can extract the leakage oscillation amplitude as $A_k(J)$, which is the largest leakage probability in all situations and therefore is independent of the explicit tomography process. In this manner we can obtain $F(J)$, shown as the red solid curve in Fig. 1(g). Evidently, $F(J)$ increases with increasing $J$ and eventually saturates.

We now consider the dephasing effect. We use $A_k'(J)$ to denote the leakage probability for the upper qubit to flip even though the lower qubit is in the |1> state in the presence of a finite qubit dephasing time. The dephasing also causes the probability for the upper (lower) pulse to flip the upper (lower) qubit, $f_U$ ($f_L$), to be less than 1. Our CNOT tomography measurement consists of four processes. We can

derive the fidelity for each process: $f_U$ for input |00>, $f_U^2 + (1 - f_U)^2$ for input |10>, $(1 - A_k') f_L$ for input |01>, and $(1 - A_k') f_U f_L + A_k' (1 - f_U) f_L$ for input |11>. The overall CNOT gate fidelity, $F'(J)$, is the minimum value among these four processes. We regard the leakage probability as $A_k'(J)$ when $W_1$ corresponds to a $3\pi$ pulse and a dephasing time of $T_2^* = 1200$ ps is considered. The value of both $f_L$ and $f_U$ is calculated to be about 0.95 for $3\pi$ pulses when $T_2^* = 1200$ ps. Finally, we obtain the process-dependent fidelity $F'(J)$, which is presented as the green dashed curve in Fig. 1(g).

$F'(J)$ exhibits fluctuations with varying $J$, especially when $J$ is small. This is because the frequency of the leakage Larmor oscillations increases with increasing $J$. If we trace a line along the $W_1$-axis in Supplementary Figure 2(e), we will inevitably encounter fluctuations in $A_k'$ with varying $J$. Despite these fluctuations, $F'(J)$ shares the same features as $F(J)$: in general, they increase with increasing $J$ and eventually saturate.

In our experiment, $J = 119$ μeV, and we obtain $F(J) = 0.97$ and $F'(J) = 0.89$. For comparison, the black dot in Fig. 1(g) represents the experimentally observed fidelity: 0.68. This observation suggests that some other effect or effects in addition to dephasing must account for the imperfect observed CNOT fidelity. We believe that the imperfect fidelity can most likely be attributed to deficiencies in the pulse shaping of the three sequential ultra-short pulses.

**Supplementary Note 3 Controlled-Universal-Rotations**

To rotate both the phases and amplitudes of the quantum states of the two qubits, we utilize the LZS interference effect [3]. As illustrated in Supplementary Figure 3(a), we apply two voltage pulses, one each to the upper and lower qubits. Both pulse widths are fixed to 100 ps, shorter than the rise and fall times combined. Therefore, the pulses can be regarded as triangular. We initialize both qubits in state |0> and choose the pulse amplitude such that it will drive each qubit through its balance point with a large sweeping velocity. We can vary either the pulse amplitude or the detuning to tune the sweeping velocity. The two qubits undergo adiabatic evolutions known as the LZS effect, meaning that both qubits rotate around the x- and z-axis in each Bloch sphere. If the two qubits are completely uncorrelated, then the LZS effect should independently transform the upper qubit from the |0> state into the $U(\beta,\psi)$ |0> + $V(\beta,\psi)$ |1> state and transform the lower qubit from the |0> state into the $U(\alpha,\phi)$ |0> + $V(\alpha,\phi)$ |1> state.

Now, let us consider the coupling between the two qubits. We apply the lower pulse first. The upper pulse follows after a delay time that is much shorter than the dephasing time. Then, the x and z rotations of the upper qubit are controlled by the resulting state of the lower qubit. Consequently, two pulses transform the two-qubit state into $U(\alpha,\phi)\, U(\beta,\psi)$ |00> + $V(\alpha,\phi)\, U(\beta,\psi)$ |10> + $V(\beta,\psi)$ |01>. The probability of finding the target in the |0> state is $P_U^0 = 1 - V^2(\alpha,\phi)\, U^2(\beta,\psi)$, and the probability of finding the control qubit in the |0> state is $P_L^0 = U^2(\beta,\psi)$. This behavior is observed in

our experiment, as shown in Supplementary Figures 3(b)-(c), where we sweep the detuning of both qubits. Theoretical simulations are presented in Supplementary Figures 3(d)-(e). The agreement between theory and experiment demonstrates that our CNOT gate functions for any quantum states of both qubits and is therefore a quantum logic gate.

This experiment demonstrates that we can rotate the phases and amplitudes of both single qubits and still achieve a CNOT gate for the combination of the two. Arbitrary quantum logic gates can, in principle, be implemented using a combination of a set of universal single-qubit gates and any one two-qubit gate such as a CNOT gate. We have demonstrated the potential of our system to serve as a universal two-qubit gate and, in principal, as a component of universal multi-qubit gates.

## Supplementary Note 4 Pulse Synchronization

The experimental ability to control one qubit by manipulating another critically relies on the precise synchronization of multiple ultra-short voltage pulses. For a CNOT gate, at most three pulses are utilized. The two pulses applied to the upper qubit are on the same gate. Therefore, their synchronization can be performed before they are fed into the fridge using a fast oscilloscope. However, the third pulse is applied to a different qubit. A time delay between the third pulse and the other two pulses will arise as they travel through different paths to reach different gates. This system time delay remains unknown until it can be measured through its effect on the two-qubit operations.

The controlled-universal-rotations, as explained in Supplementary Note 3, can be utilized to synchronize the pulses on the upper and lower gates. We apply a 100-ps pulse to the control qubit and another 100-ps pulse to the target qubit. We must use ultra-short pulses for precise determination of the system time delay. The effect is that the rotation of the upper qubit is controlled by the rotation of the lower qubit, under the assumption that the upper pulse immediately follows the lower pulse. If the upper pulse comes too late after the lower pulse has finished, then qubit dephasing will cause the two qubits to behave independently.

Conversely, if the lower pulse comes immediately after the upper pulse, then the rotation of the lower qubit will be controlled by the upper qubit. If the lower pulse arrives too late compared with the upper qubit, then the rotation of both qubits will again be independent.

We modify the time delay between the pulses applied to the upper and lower qubits at the pulse generator. The unknown system time delay along the transmission path is then added to this predetermined delay to yield the final time delay. We record the coherent rotations of both qubits under these pulses, as shown in Supplementary Figures 4(a)-(f). We conclude that the system time delay is approximately +200 ps (the positive sign means that the upper pulse lags behind the lower pulse by 200 ps), because a -200 ps predetermined delay just cancels the system delay and yields

independent coherent rotations for the two qubits. This number has been applied to all experiments presented in this paper.

**Supplementary References**

1 Dohun Kim, et al. Quantum control and process tomography of a semiconductor quantum dot hybrid qubit, Nature 511, 70-74 (2014).

2 K. D. Petersson, et al. Quantum coherence in a one-electron semiconductor charge qubit. Phys. Rev. Lett. 105, 246804 (2010).

3 G. Cao, et al. Ultrafast universal quantum control of a quantum-dot charge qubit using Landau-Zener-Stuckelberg interference. Nature Commun. 4, 1401 (2013).
